# Microbial degradation of low-density polyethylene (LDPE) by *Aspergillus clavatus* strain JASK1 isolated from landfill soil

**DOI:** 10.1007/s13205-016-0394-x

**Published:** 2016-02-13

**Authors:** Anudurga Gajendiran, Sharmila Krishnamoorthy, Jayanthi Abraham

**Affiliations:** Microbial Biotechnology Laboratory, School of Biosciences and Technology, VIT University, Vellore, Tamil Nadu 632014 India

**Keywords:** Microbial degradation, LDPE films, Strain JASK1, Strum test, FTIR, SEM

## Abstract

Polythene and plastic waste are found to accumulate in the environment, posing a major ecological threat. They are found to be considered non-degradable, once it enters the environment it has been found to remain there indefinitely. However, significant attention has been placed on biodegradable polymer, identification of microbes with degradative potential on plastic material. The aim of the present investigation was to biodegrade low-density polyethylene (LDPE) using potential fungi isolated from landfill soil. Based on 18S rRNA analyses the isolated strain was identified as *Aspergillus clavatus*. LDPE degradation by *A. clavatus* was monitored for 90 days of incubation in aqueous medium. The degradation was confirmed by changes in polyethylene weight, CO_2_ evolution by Strum test, infrared spectra and morphological changes by SEM and AFM analysis.

## Introduction

During the past decade polyethylene materials have gained widespread use in various fields and have become indispensables. They offer a number of advantages over other materials being versatile, light weight, low cost, strong and potentially transparent and are ideally suited for a variety of applications. However, plastic materials have several disadvantages, the most important criterion is its long-term persistence in the environment and their resistance to degradation. A general estimate of worldwide plastic waste generation is about 57 million tons (Kumar et al. 2007). Polyethylene is the one of the most abundant commercially produced synthetic polymers. LDPE accounts for 60 % of the total plastics production of plastic bags and most commonly found solid waste. Polyethylene is very resistant to biodegradation due to its high hydrophobicity and its long carbon chains (Contat-Rodrigo and Ribes Greus 2002). Under normal condition, it takes more than 10 decades to mineralize the polymers (Ohtake et al. 1998). Several cities in Brazil have prohibited the distribution of plastic bags by supermarkets and other commercial establishments because they are made of various chemicals which are toxic to the health and the environment. An estimated one million birds and ten thousands marine animals die each year as a result of ingestion of plastics (Palmisano and Pettigrew 1992). Intensive research is being carried by scientist in the development of degradable plastics to enhance biodegradability of plastic products in landfills and composts. Degradable plastic is a plastic designed to undergo significant changes in its chemical structure under specific environmental conditions resulting in loss of some of its properties (Albertsson et al. 1987). Polymeric characteristics such as molecular weight, crystallinity, functional groups, mobility, substituent present in the structure and the additives added to the polymers play a significant role in its degradation (Gu et al. 2000). The fate of these organic polymers in the environment and the time required for their total mineralization to CO_2_ have yet to be fully understood. To relieve the abnormal circulation, there are two ways, one is to develop the latent ability of microorganisms to degrade plastics presently used and the other is to develop synthetic polymers susceptible to degradation (Hiroyuki et al. 1978). Low-density polyethylene can be degraded in various methods as follows: chemical degradation, photodegradation and biological degradation (Da Luz et al. 2014). The microbial species associated with the degrading polymers were identified as bacteria (*Pseudomonas*, *Streptococcus*, *Staphylococcus*, *Micrococcus)*, *fungus (Aspergillus niger*, *Aspergillus glaucus* and *Trichoderma*) (Swift 1997). There are many researchers around the globe investigating the ability of enzymes released by microbes in oxidizing the hydrocarbon of polymer. Microbial enzymes induce the rate of biodegradation of LDPE very effectively without causing harm to the environment. The extracellular enzymes are too large to penetrate deeply into the polymer material, and so, act only on the polymer surface, consequently the biodegradation of plastics is usually a surface erosion process. The UV irradiation (photo-oxidation) and thermal and chemical oxidation of polyethylene prior to its exposure to a biotic environment enhances biodegradation.

The aim of this investigation is to evaluate the biodegradation of LDPE films by exposing them to the isolated fungal strain. Fungus was chosen as they are highly diverse and they play a major role in decomposition of lignocellulose polymer and posses wider metabolic capabilities. The morphological changes of LDPE were analyzed after degradation through AFM and SEM.

## Materials and methods

### Chemicals

The chemicals used in this present study were purchased from Sigma Aldrich (St. Louis, MO, USA). All other reagents used in this study were of high purity and analytical grade.

### Polyethylene

The LDPE films used in this study were collected from Vellore market, which were 20-µm-thick bags. For the biodegradation studies, LDPE films were cut into small strips and were sterilized with 70 % ethanol.

### Sample collection

Landfill soil was collected from Vellore Institute of Technology (VIT), Tamil Nadu, India and was used for the isolation of LDPE-degrading microorganisms.

### Isolation of microorganisms

The soil sample collected was homogenized and passed through a 2-mm sieve to remove gravel. One gram of soil sample was added to 99 ml of sterile double distilled water. The soil solution was shaken properly and serially diluted. For each dilution triplicate potato dextrose plate was made to isolate fungus. Plates were incubated at 25 °C for 48 h. The developed colonies were isolated and sub-cultured repeatedly to obtain the pure culture and preserved in slants at 4 °C.

### Screening of polythene-degrading fungus

Enrichment technique was carried out to select the efficient polythene-degrading fungus. M1 broth was used to enrich fungus. 100 ml of M1 medium containing (g l^−1^) NaNO_3_ 2; KCl 0.5; MgSO_4_·7H_2_O 0.5; glucose 10; FeCl_3_ 10; BaCl_2_ 0.2 and CaCl_2_ 0.5 at pH 6.8 were supplemented with 0.5 g of plastic strips (2 × 2 cm), respectively, as sole source of carbon and energy. One ml of isolated cultures was inoculated in respective flasks. These flasks were incubated in a shaker at 120 rpm for 15 days. After three cycles of enrichment, one ml of the sample was serially diluted and plated on PDA plate for isolation of fungus. The morphological identification of the fungal isolates was done by performing lacto phenol cotton blue staining.

### Taxonomic identification of fungal strain

The isolated fungal strain was identified by 18S rRNA sequence analysis. The fungal genomic DNA was isolated using AMpurE Fungal gDNA Mini kit (Amnion Biosciences Pvt. Ltd. Bangalore, India). In this kit, detergent and other non-corrosive chemicals are used to break open the cellulosic cell wall and plasma membrane to extract DNA from fungal cells. The 18S rRNA gene was amplified by polymerase chain reaction (PCR) using the universal primers 5′-CGWCGRAANCCTTGTNACGASTTTTACTN-3′ and 5′AWGCTACSTGGTTGATCCTSCCAGN-3′. PCR mix of 50 µl final volume contained: 50 ng sample gDNA, 100 ng forward primer, 100 ng reverse primer, 2 µl dNTPs mixture (10 mm), 5 µl 10X Taq polymerase buffer, 3 U Taq polymerase enzyme and PCR grade water to make up the volume. Amplified PCR product was sequenced using ABI3730xl genetic analyzer (Amnion Biosciences Pvt. Ltd. Bangalore, India). The sequencing result was submitted to the Genbank National Centre for Biotechnology Information (NCBI) database. Multiple alignments of partial sequence were performed by CLUSTALW and the phylogenesis was analyzed using MEGA 4.0 software. An unrooted tree was built using the neighbor-joining method (Tamura et al. 2007).

### Biodegradation studies

#### Determination of dry weight of residual LDPE

For the accurate measurement of dry weight of residual LDPE, the LDPE films were recovered from the degradation medium and they were washed with 2 % (v/v) sodium dodecyl sulfate (SDS) solution and further rinsed with distilled water (Gilan et al. 2004). The washed LDPE film was dried overnight at 60 °C before weighing and the percentage of weight loss was determined using the formula (Kyaw et al. 2012):

$$ {\text{Weight loss}}\;(\% ) = \frac{{{\text{Initial weight}} - {\text{Final weight}}}}{\text{Initial weight}} \times 100 $$

### CO_2_ evolution

CO_2_ evolved as the result of LDPE degradation was determined gravimetrically by Strum test. LDPE strips were added to the test flask containing 100 ml of enrichment medium. The LDPE was incubated with *Aspergillus clavatus* which served as the test and LDPE without the fungal strain served as the control. Both the flasks were incubated at room temperature for 4 weeks. After incubation, both the metabolic and atmospheric CO_2_ from the test flask and atmospheric CO_2_ from the control flask were calculated gravimetrically. The CO_2_ evolved, as a result of degradation of polymeric chain was trapped in absorption flask containing KOH (1 M). Barium chloride solution (0.1 M) was added to the flask containing KOH and which resulted in barium chloride precipitation (using CO_2_ released from the breakdown of polymer). CO_2_ produced was calculated gravimetrically by measuring the amount of CO_2_ evolved by addition of BaCl_2_. Changes from that of control were recorded.

### Spectroscopic analysis

The changes in the polymer bonds due to biodegradation of *A. clavatus* strain JASK1 were determined using FTIR spectrophotometer (8400 Shimadzu, Japan, with Hyper IR-1.7 software for Windows). The LDPE film exposed to the isolates was analyzed after 90 days of incubation period which was recorded from frequency of 4000–400 cm^−1^ at a resolution of 4 cm^−1^ at room temperature with a helium–neon laser lamp as a source of IR radiation.

#### Atomic force microscopy (AFM)

Modifications on the surface topography of the treated and untreated LDPE film were examined by AFM (Nanosurf 2 Easyscan). The degraded LDPE film samples along with the control LDPE film were analyzed in a scan speed of 1.0 Hz and a resolution of 512 × 512 pixels. For the AFM studies polythene films were recovered from the respective media and the films were washed with 2 % sodium dodecyl sulfate to remove adhered microbes. Thereafter, the film was air-dried overnight and was used for analysis (Tribedi and Sil 2013).

#### Scanning electron microscopy (SEM)

The treated samples after 90 days of incubation with *A. clavatus* was subjected to SEM analysis after washing with 2 % (v/v) aqueous SDS and distilled water for few minutes and flushed with 70 % ethanol to remove the cells. The sample was pasted onto the SEM analysis stub using a carbon tube and the sample was coated with the gold for 40 s and analyzed under high-resolution scanning electron microscope (EVO LS15; Carl Zeiss, Germany).

## Results and discussion

### Isolation and identification of LDPE-degrading strain

**Fig. 1:**
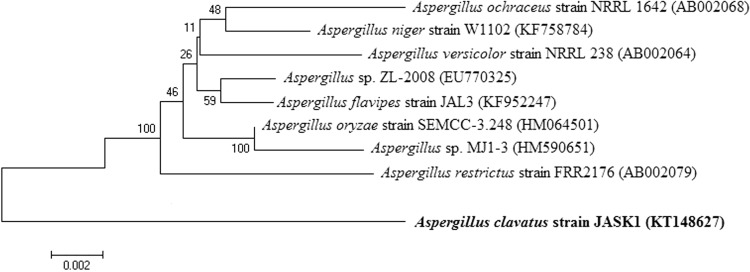
Phylogenetic relationship of *Aspergillus clavatus* JASK1 based on 18S rRNA gene nucleotide sequences. Numbers at the nodes indicate bootstrap values from the neighbor-joining analysis of 1000 resampled data sets. *Bar* represents sequence divergence

All the fungal isolates from landfill soil area were screened for their potency to degrade LDPE after 8–10 days of incubation time at 25–30 °C in enrichment medium. Based on this screening, strain JASK1 was selected for LDPE degradation studies. The 18S rRNA nucleotide sequence of JASK1strain was deposited in the NCBI and Gene bank and the accession number KT148627 was obtained. The sequences with the highest 18S rRNA partial sequence similarity were selected and compared by CLUSTAL W. Phylogenetic and molecular evolutionary analyses were conducted by MEGA 4.0 software with the Kimura 2-parameter model and the neighbor-joining algorithm. The alignment of these sequence with other sequence found in the data base showed a 99 % similarity with the sequence of *A. clavatus* JASK1strain (Fig. 1).

A previous study conducted by Kathiresan (2003) on soil microbes revealed their active association in biodegradation of plastics and polyethylene bags. Priyanka and Archana (2011) made a comparative analysis between the biodegradation of polythene and plastic by five different types of soil sample collected from different sources. Among various species of bacteria and fungus, *Bacillus subtilis*, *A. niger*, *Aspergillus nidulance*, *Aspergillus flavus*, *Aspregillus glaucus*, *Penicillium*, *Pseudomonas*, *Staphylococcus aureus*, *Streptococcus lactis*, *Proteus vulgaris*, *Micrococcus* were found to degrade polythene and plastic efficiently (Abrusci et al. 2011; Aswale and Ade 2008; Kathiresan 2003; Nanda et al. 2010; Reddy 2008). Esmaeili et al. (2013) reported that *Aspergillus* sp., and *Lysinibacillus* sp., isolated from landfill soil sample were able to degrade LDPE efficiently.

### Biodegradation studies

After incubation of 90 days, the degrading capacity of the strain JASK1 was analyzed using various parameters and the results were interpreted.

### Weight reduction

**Fig. 2:**
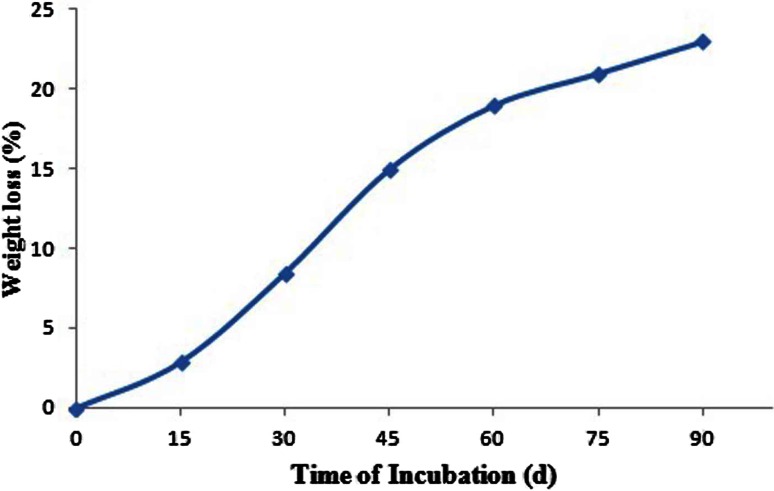
Graph represents the percentage weight loss of LDPE films incubated with *Aspergillus clavatus* JASK1

A simple and quick way to measure the biodegradation of polymers is by determining the weight loss. Microbes that grow utilizing the polymer lead to an increase in weight due to the adherence of microbes, whereas a loss of polymer integrity leads to weight loss. Weight loss of LDPE is proportional to the surface area since biodegradation usually is initiated at the surface of the polymer. After the degradation period, the LDPE films were treated with SDS as surfactant which denatures the cells and completely wash off from the surface. The reduction in weight was observed after the biodegradation of LDPE. In our study, 35 % weight loss of LDPE films was observed after 90 days of incubation with *A. clavatus* strain JASK1 whereas in control flask there is no weight loss of LDPE films. The experiment was conducted thrice and the percentage changes in the weight of LDPE from 15 to 90 days of incubation with strain JASK1 are shown in Fig. 2. Raaman et al. (2012) conducted the study on biodegradation of LDPE under laboratory condition, they isolated *A. niger*, *A. japonicas*, *A. terreus*, *A. flavus* and *Mucor* sp. from polluted soil sample, among these strains *Aspergillus japonicas* efficiently degraded 11.11 % of LDPE in 1 month of incubation time and *A. niger* degraded 5.8 % in 1 month.

### CO_2_ evolution

Strum test was followed to assess degradation of metabolic carbon dioxide evolved during the growth period. In the present study, the LDPE was incubated for 4 weeks, along with *A.* *clavatus* sp. resulting in 2.32 g l^−1^ production of CO_2._ The findings were found to be similar with the work done by Shah et al., (2009) who reported 1.85 g l^−1^ evolution of CO_2_ after a 30 day period of incubation with fungal strain of *Fusarium* sp. on LDPE films. Similar results were also recorded by Das and Kumar (2014) with CO_2_, CH_4_ and H_2_O as end products of polyethylene degraded by *Fusarium* strain.

### FTIR analysis

**Fig. 3:**
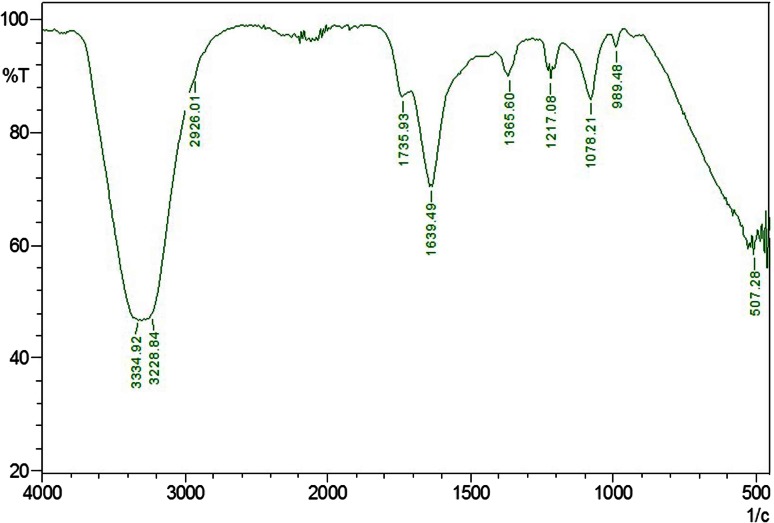
FTIR spectrum of biodegradation of LDPE film after 90 days of incubation. The presence of functional groups supporting the conformational change on the polymer surface

The structural analysis is the important parameter to identify the structural changes which appear during degradation responsible for weight loss. FTIR is sensitive to local molecular environment and as a consequence has been widely applied to investigate the interactions between the macromolecules during LDPE degradation. FTIR analysis of the degraded LDPE films gives a close view of N–H stretching of aldehyde group at 3334.92 and 3228.84 cm^−1^, C–C=C symmetric of aromatic ring at 1639.49 cm^−1^, C=O stretching of aldehyde group at 1735.93 cm^−1^, peak at N=O bend which corresponds to 1365.60 cm^−1^, C–O stretching of ether group at 1217.08 and 1078.21 cm^−1^. The most prominent structural changes were observed in the LDPE sample degraded by strain JASK1 depicted in Fig. 3.

Our findings are found to be consistent with the study of Esmaeili et al. (2013) where the spectra of the film incubated in soil showed several new bands. The carboxyl absorption band was evident at 1710–1750 cm^−1^ due to the formation of ketone or aldehyde C=O groups by the microorganisms. Similar pattern was also observed by Da Luz et al. (2014) where the oxo-biodegradable plastic by *Pleurotus ostreatus* was analyzed through FTIR spectrum. Koutny et al. (2006) and Sudhakar et al. (2008) examined the same results in their study. Das and Kumar (2014) noticed the formation of new and disappearance of functional group in their LDPE degradation studies by *Bacillus amyloliquefaciens* strain. The changes in the peak values of almost all functional groups support the conformational change on LDPE sample.

### AFM analysis

**Fig. 4:**
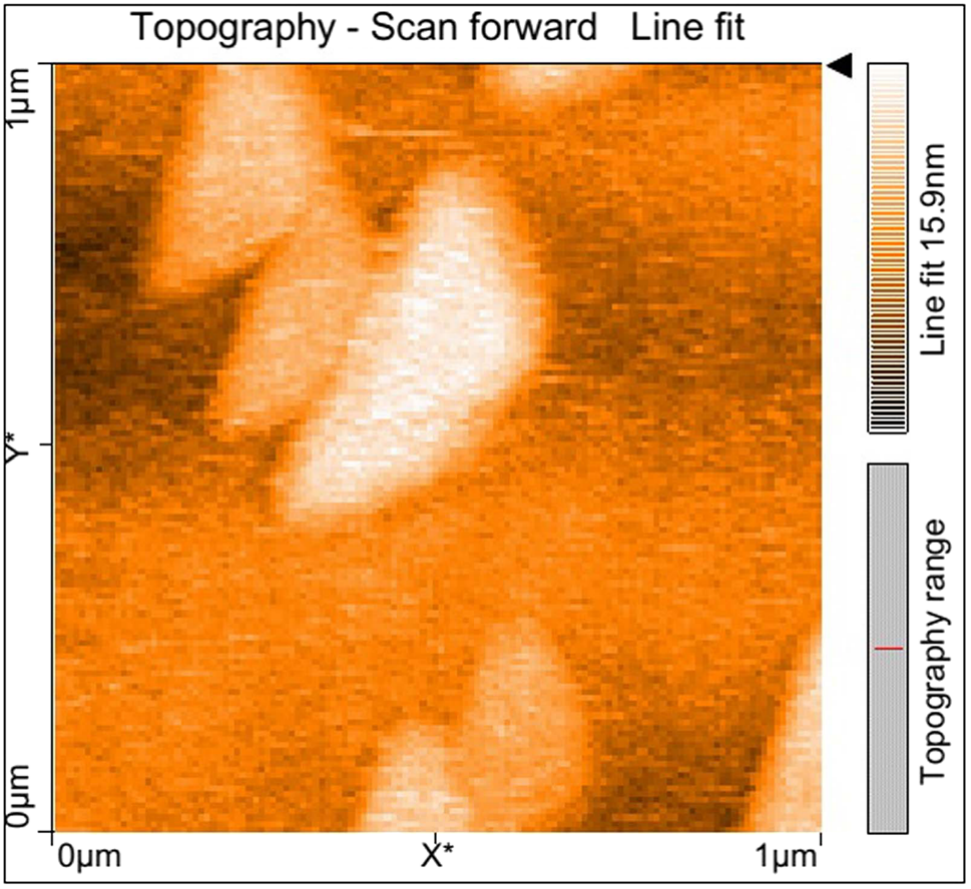
AFM micrographs revealing the surface changes of LDPE films after 90 days of incubation with *Aspergillus* *clavatus*

The AFM micrographs demonstrate localized degradation of the LDPE in the presence of *A. clavatus* strain JASK1 resulting in the formation of grooves, fractures and mild erosion (Fig. 4). The isolate *A. clavatus* strain JASK1 was found to degrade LDPE even without pre-treatment. In a study conducted by Esmaeili et al., (2013) after incubation of LDPE with *A. niger* for 126 days there was formation of pits and cavities on the surface, which suggested that the fungus had penetrated into LDPE matrix during the degradation period.

### Scanning electron microscopy analysis

**Fig. 5:**
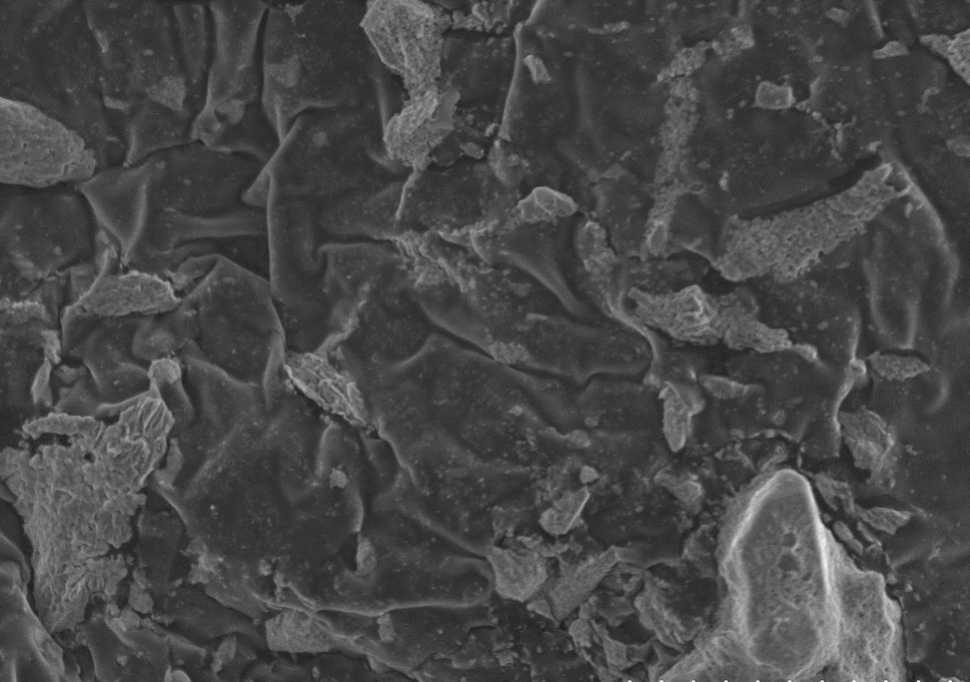
Scanning electron microscopy of *Aspergillus* *clavatus* JASK1 growing on the surface of LDPE films after 90 days of incubation showing the physical weak, minor cracks and wrinkles on the surface of LDPE films

SEM analysis is used to confirm that the surface of LDPE becomes physically weak after biological treatment. After 90 days of incubation, fungal colonization was observed on the surface of LDPE by SEM. SEM experiment has shown the presence of fungus on the surface of the LDPE. LDPE films exposed to *A. clavatus* strain JASK1 showed surface erosion, cracks, folding and fungal colonization (Fig. 5). This may be due to the extracellular metabolites and enzymes released by the fungus in response to stress. Surface bio-erosion is the primary cause of mass loss from surface. As a cross reference to the earlier studies on the biodegradation of LDPE, many researchers have reported the same morphological changes on LDPE degradation by *Aspergillus* (Volke-Sepulveda et al. 2002; Limon-Gonzalez and Favela-Torres 2004; Sahebnazar et al. 2010).

## Conclusion

The research article deals with efficient degradation of LDPE by the isolate *A. clavatus* for duration of 90 days exposure. To the best of our knowledge, there are no research articles supporting polyethylene degradation by *A. clavatus.* *A. clavatus* qualifies as a suitable candidate for LDPE degradation. As LDPE accumulation in the environment is a serious threat this isolate will be of major use in degradation. Though there are many works on LDPE degradation there is no detailed explanation on molecular mechanism behind degradation which will help in implementing biodegradation of LDPE.
